# Esophageal metastases from primary lung cancer: a case report

**DOI:** 10.1186/s13256-021-02765-x

**Published:** 2021-05-12

**Authors:** Chang-Yong Wang, Gang Xu, Chuan Gao, Dong Wang

**Affiliations:** 1 Department of Cardiothoracic Surgery, Jinling Hospital, Nanjing, 210000 Jiangsu China; 2 Department of Cardiothoracic Surgery, Affiliated Taikang Xianlin Drum Tower Hospital, Medical School of Nanjing University, Nanjing, 210000 Jiangsu China

**Keywords:** Lung cancer, Esophageal metastases, Case report

## Abstract

**Background:**

Primary lung cancer is one of the most frequently diagnosed cancers. The common metastatic sites are the liver, bones, brain, adrenal glands and central nervous system. However, gastrointestinal metastases, particularly esophageal metastases, from lung cancer are rare. There are no cases of esophageal metastases from lung cancer which refer to its particular treatment.

**Case presentation:**

We report a case of esophageal metastases from lung cancer. The patient was a 55-year-old Han Chinese man who first attended our hospital due to dry cough and was diagnosed with late-stage lung cancer. Three months later, the patient complained of dysphagia. Endoscopic ultrasonography (EUS) and pathological examination of the biopsy specimen was performed to confirm the lesion was metastases from lung cancer. Thyroid transcription factor 1 (TTF-1), cytokeratin 7 (CK-7) and napsin A were positive by immunohistochemistry examination. These results reconfirmed the diagnosis of esophageal metastases from lung cancer.

**Conclusions:**

Esophageal metastasis from lung cancer is very rare. It may be alleviated with personalized chemotherapy. In addition, molecular targeted therapy for patients with epidermal growth factor receptor (EGFR) mutations may be reasonable.

## Background

Lung cancer is a major cause of cancer-related death worldwide [1]. When diagnosed, approximately 50% of patients present with metastases. The most common metastatic sites are the liver, bones, brain, adrenal glands and central nervous system [2]. However, gastrointestinal metastases, particularly esophageal metastases, from lung cancer are rare [3]. Furthermore, most cases of esophageal metastases are asymptomatic, and are finally discovered at autopsy [4] rather than in clinics.

Here we report a case of primary lung cancer metastasized to the esophagus and discuss diagnostic and treatment strategies, in order to give other doctors some supplemental information and references when treating such uncommon patients.

## Case presentation

**Fig. 1:**
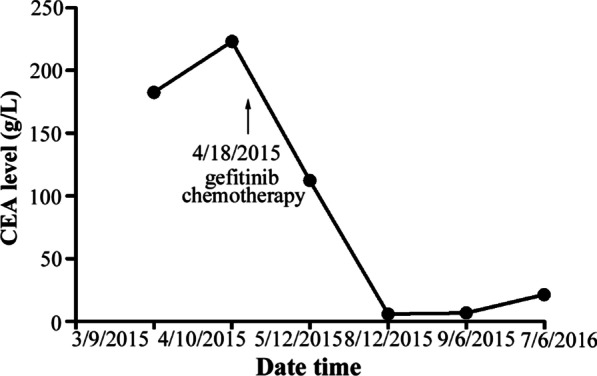
Alteration of carcinoembryonic antigen (CEA) level and treatment at different time points. The patient first presented with an increased CEA level. The carcinoembryonic antigen level gradually decreased to 6.7g/L (normal value, 0–9.8 g/L) following 5 months of gefitinib and chemotherapy treatment

**Fig. 2:**
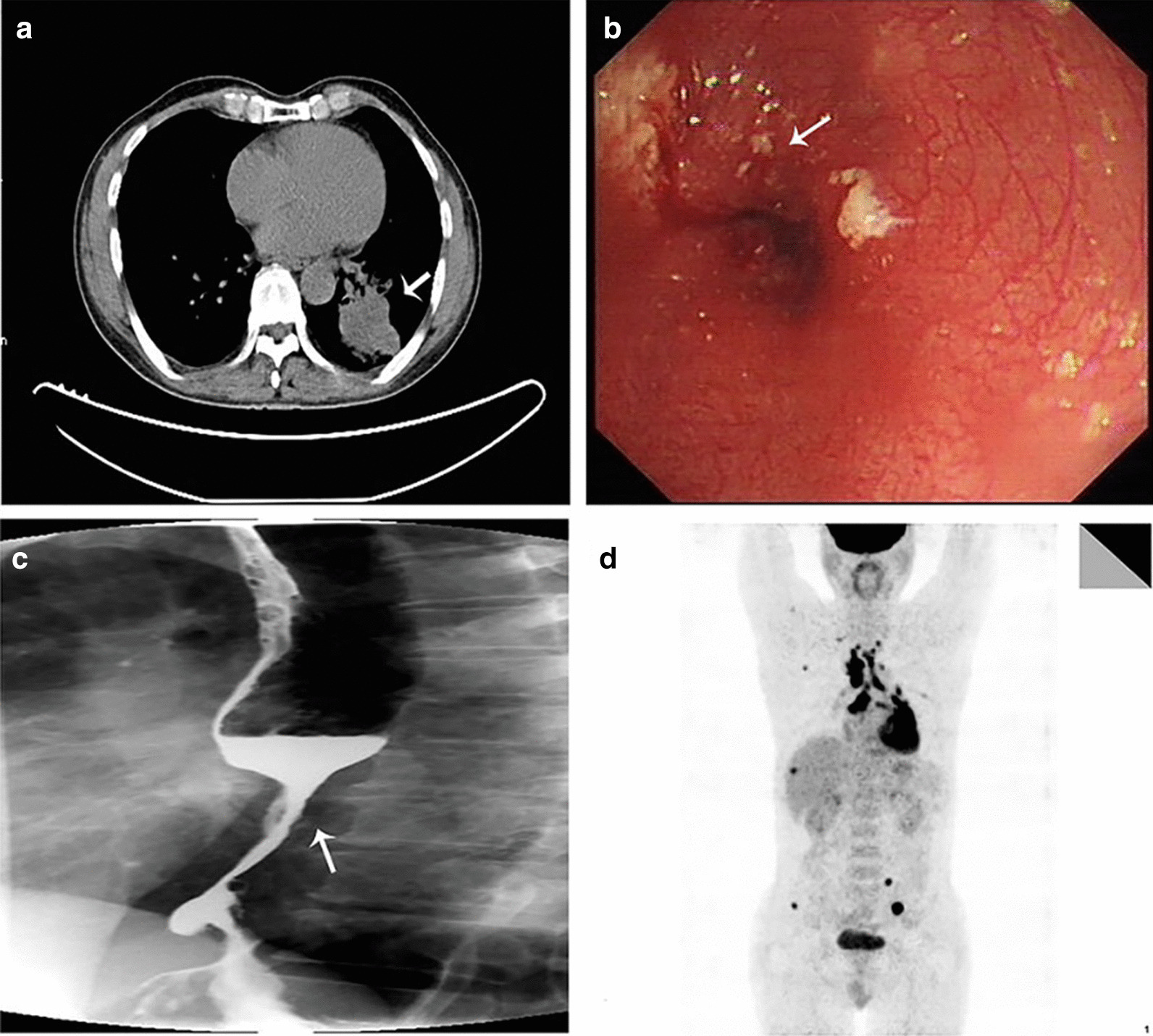
Imaging study of a patient with esophageal metastases from lung cancer. **a** Computed tomography (CT) scan of the chest showed a 48 × 42 mm defined tumor at the ascending left lung (arrow), with a scallop-shaped contour and focal enhancement. **b** Gastroscopy images showed a 17.7 mm wall thickening at the distal esophagus 38 cm from the upper incisors (arrow), with the mucous epithelium being mildly hemorrhagic but without a distinct break. **c** Barium esophagography. Esophagography showed irregular thickening of the wall and narrowing of the lumen of the lower thoracic esophagus (arrow). **d** Positron emission tomography (PET)-CT. PET-CT showed multiple metastases (lower left lung, bilateral supraclavicular fossa, hilus pulmonis, mediastinum lymph node, bone).

**Fig. 3:**
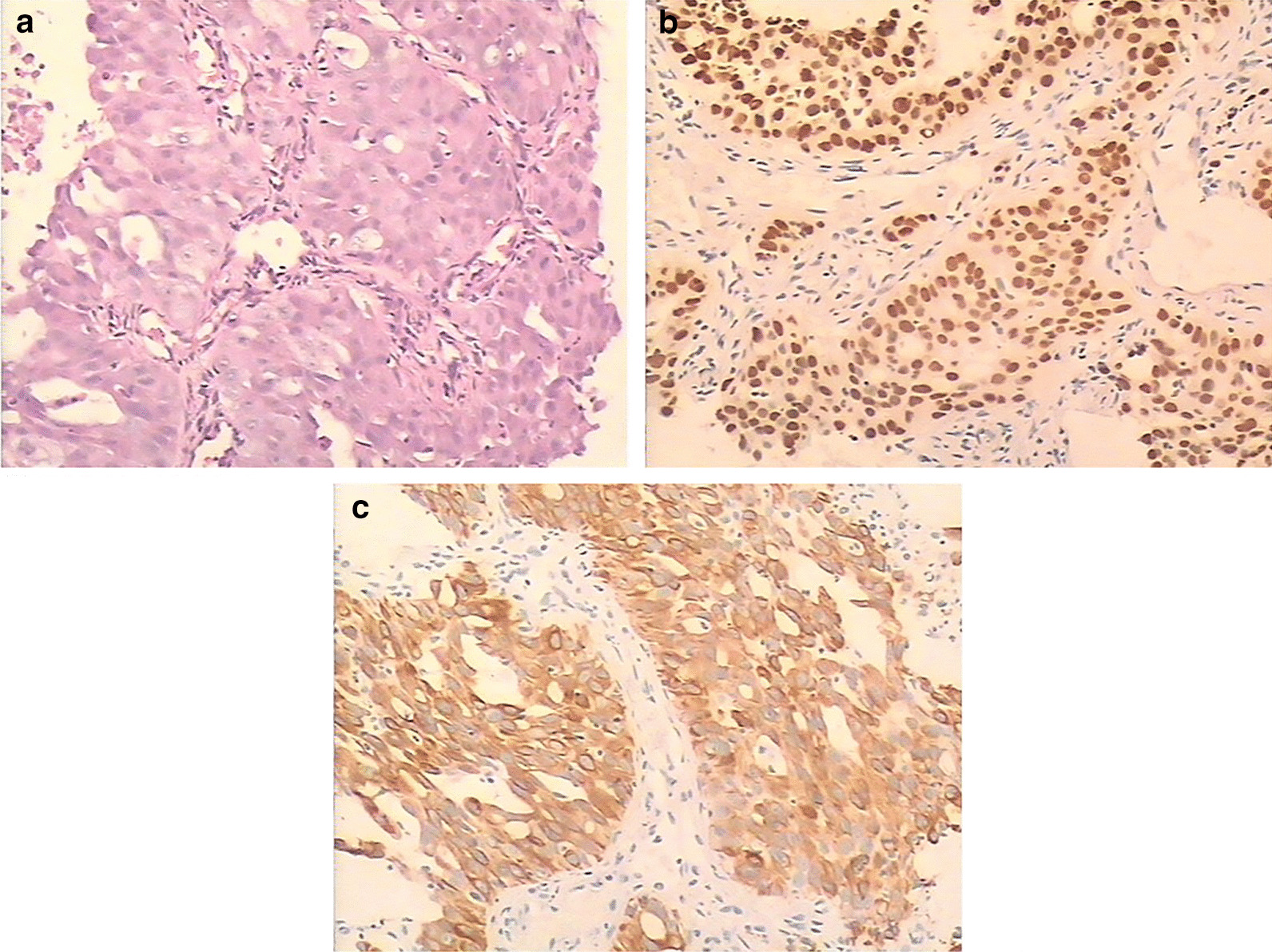
Histological and immunohistochemical staining results. **a** Cancer tissue revealing the atypical nest shape of the cell arrangement (stain, hematoxylin and eosin [HE]; magnification, ×100). The positive immunohistochemical staining (magnification, ×200) for **b** TTF-1 and **c** CK-7 indicates that the cancer originated in the lung. TTF-1 is specific for tumors of lung origin. TTF-1 (thyroid transcription factor 1); CK (cytokeratin)

A 55-year-old Han Chinese man with a long-term history (30 years) of heavy smoking was referred to our hospital in March 2015 due to dry cough. His past medical history and family history were unremarkable. On a routine health checkup, an elevated carcinoembryonic antigen (CEA) value of 182.6 g/L (normal value, 0–9.8 g/L; Fig. 1) was found. A computed tomography (CT) scan of the chest showed a 48 × 42 mm tumor in the left lower lobe (Fig. 2a). A positron emission tomography (PET)-CT scan revealed multiple metastases in the left lower lung, bilateral supraclavicular fossa, hilus pulmonis, mediastinal lymph nodes and bone (Fig. 2d). The patient underwent CT-guided biopsy of the lung lesion. The biopsy tissue was identified as adenocarcinoma by pathological examination (Fig. 3a). Immunohistochemistry (IHC) examination showed that the tumor cells were positive for thyroid transcription factor 1 (TTF-1) and cytokeratin 7 (CK-7) (Fig. 3b, c). The epidermal growth factor receptor (EGFR) gene of the primary lung cancer harbored a mutation of the 19th exon. Clinically, the patient was diagnosed with stage IV lung adenocarcinoma, with the primary lesion in the left lower lobe. After communicating with the patient and his family, gefitinib (250 mg once a day) was initially commenced in April 2015. The primary tumor shrank about one-fifth after 2 months of treatment, and the patient did not experience obvious adverse effects.

**Fig. 4:**
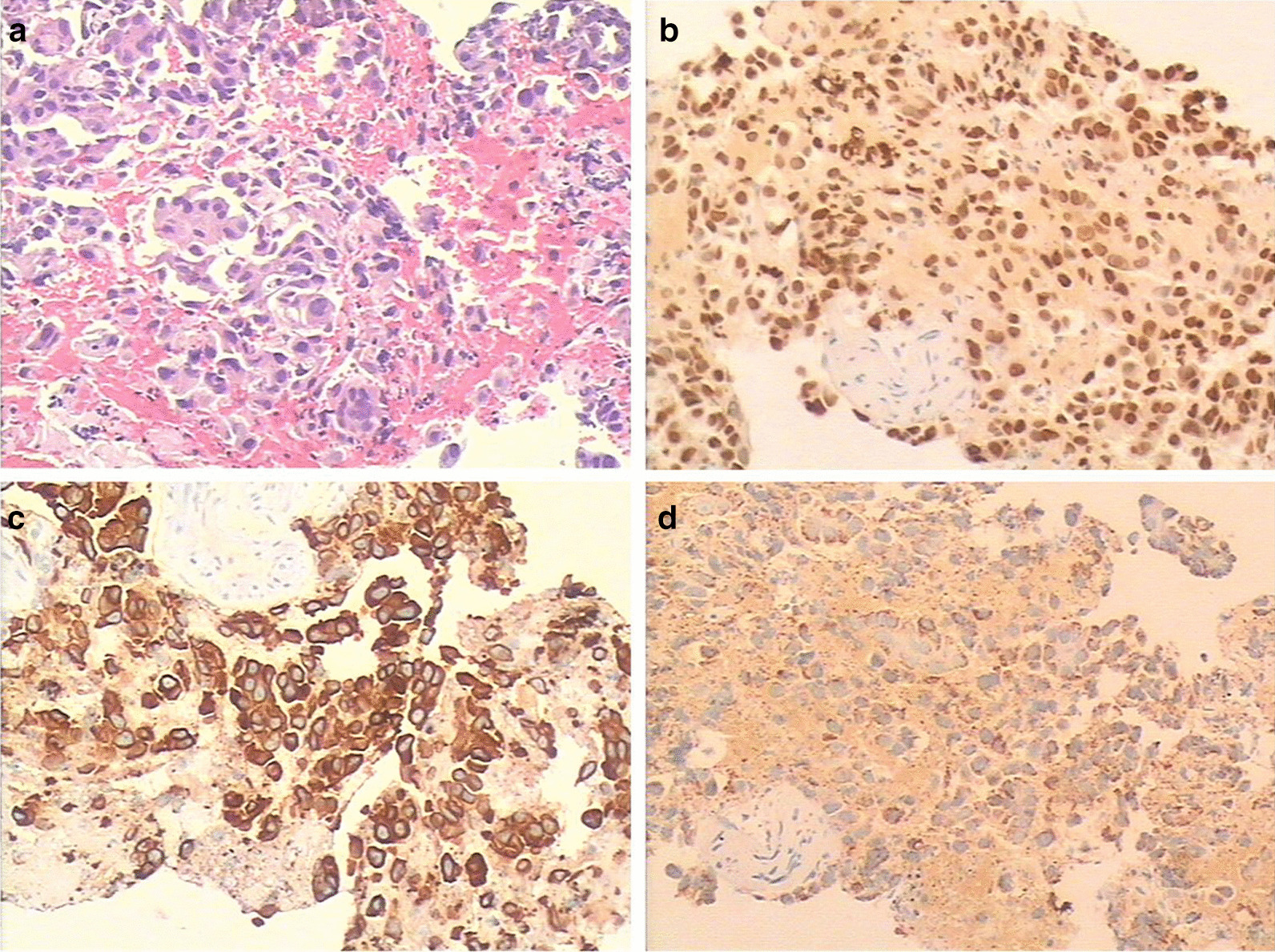
Histological and immunohistochemical staining results. **a** Hematoxylin and eosin (HE) staining of the esophageal lesions (magnification, ×100). The cancer tissue exhibits similar HE morphology as lung adenocarcinoma. Positive immunohistochemical staining (magnification, ×100) for **b** thyroid transcription factor 1 and **c** cytokeratin 7, which is the same result as found in the lung adenocarcinoma. **d** Napsin A of the core biopsy of the esophageal mass shows stippled cytoplasmic positive staining. Napsin A is specific for tumors of lung origin

Three months later, during follow-up, the patient complained of dysphagia without melena. The patient could only eat fluids. Laboratory examinations indicated a an elevated CEA level of 112.3 g/L. Barium esophagography showed irregular thickening of the wall and narrowing of the lumen of the lower thoracic esophagus (Fig. 2c). Endoscopic ultrasonography (EUS) showed a 17.7 mm wall thickening at the distal esophagus 38 cm from the upper incisors, with mucous epithelium being mildly hemorrhagic but without a distinct break (Fig. 2b). There was also no evidence of active bleeding. The patient underwent biopsy of the esophageal lesion tissue under endoscopic ultrasonography (EUS). Pathological examination of the esophageal lesion tissue showed similar results as those of the primary lung adenocarcinoma (Fig. 4a). TTF-1 and CK-7 were positive by IHC (Fig. 4a, b). Napsin A staining was positive (Fig. 4d). Brain magnetic resonance imaging (MRI) revealed multiple metastases. The patient then received additional chemotherapy with docetaxel (60 mg day 1, day 8) and cisplatin (20 mg days 1–5), with gefitinib terminated. After one cycle of chemotherapy, the patient complained of chest tightness after activity. A CT scan showed pleural effusion and progression of the primary tumor. Closed thoracic drainage and injections of lentinan and cisplatin (two cycles) into the chest were conducted to alleviate the patient’s symptoms. The chemotherapy was then adjusted to pemetrexed (0.8 g day 1) and cisplatin (20 mg days 1–5). After six cycles of chemotherapy, the patient gradually returned to a normal diet. Follow-up CT scans showed complete response in the esophageal lesion, and the CEA level decreased to 6.7 g/L in September 2015. The primary lung tumor had partially shrunk. The patient then continued to take gefitinib (250 mg once daily), and the primary tumor was stable. In June, 2016, the patient began having headaches. Brain MRI revealed that the metastases had progressed. The CEA level rebounded to 21.3 g/L. The patient then received AZD9291 orally (80 mg once daily) without T790m mutation testing. His headache symptoms were alleviated, and there was no further follow-up of the patient. We learned by telephone follow-up that the patient died in January 2017.

## Discussion

This case reported a patient who was diagnosed with primary lung cancer with multiple metastases at the first visit, and the tumor metastasized to the esophagus during treatment. We recorded this case not only because it is a rare case in clinics, but also because of the many clinical characteristics during the whole course of treatment.

Lung cancer typically spreads to the liver, bones, brain, adrenal glands and central nervous system [4]. However, according to autopsy studies, the overall incidence of esophageal metastasis in patients dying of any type of cancer is approximately 3–6%, among which breast and lung represent the most common sites of the primary tumor [3].

There are several possible routes by which lung cancer can metastasize to the esophagus: bloodstream and lymphatic metastases, implantation metastases and direct invasion from nearby organs, namely the larynx, hypopharynx, trachea, bronchus, stomach and mediastinal lymph nodes [5]. According to the almost normal overlying mucosa observed by endoscopy in our patient’s esophagus, we consider that the metastases came from the bloodstream or lymphatic route, as this mechanism leads mainly to submucosal lesions.

Most patients with metastases to the esophagus have no specific symptoms. Even when symptoms are present, they are usually nonspecific. Only a small number of patients complain of dysphagia, hoarseness and weight loss [6]. Our patient showed dysphagia due to stricture as a result of lymphatic or bloodstream metastases. Esophagography and endoscopy are useful diagnostic tools. Esophagram demonstration is characterized by regular stenosis of the esophagus. However, endoscopy often shows a normal mucosal surface when there is no serious growth of the esophageal metastasis. With the help of endoscopic ultrasound (EUS), more accurate information will be provided about the submucosal lesion [7]. Biopsies guided by EUS will reveal pathological findings. Histological and immunohistochemical staining can provide crucial information to distinguish whether the esophageal lesion comes from the lung adenocarcinoma. TTF-1 is specific for tumors of lung origin. TTF-1 is expressed in the epithelial cells of the thyroid gland and lung, while adenocarcinomas are malignant transformations evolved from these cells [8]. TTF-1 has been shown to be a sensitive and highly specific marker of adenocarcinomas of pulmonary origin, in tissue biopsies and cytological preparations. TTF-1 is expressed mainly in 68–80% of primary lung adenocarcinomas, but expressed in less than 1% of adenocarcinomas of non-pulmonary origin [9, 10]. Napsin A is also specific for tumors originating from the lung. Napsin A is expressed in 84.5% of primary lung adenocarcinomas, but not expressed in adenocarcinomas of other sites. Double staining of TTF-1 and napsin A has been proposed to increase the sensitivity and specificity of pulmonary-originated adenocarcinomas. [11]. Moreover, CK-7 can identify primary lung cancer. However, some studies found that primary adenocarcinomas of the rectum and small intestine may also express CK-7 in a small number of cases [12]. In our case, due to the marker’s high specificity, positive results for TTF-1 and napsin A provided strong evidence of the pulmonary origin of the esophageal lesions, with addition of the clinical and morphological findings.

Accurate distinction between esophageal metastasis and the primary cancer is of key importance, as it determines the treatment and prognosis of patients [4]. Once the patient was diagnosed with esophageal metastasis originating from the pulmonary tumor, we administered chemotherapy, which achieved good results in the esophageal metastasis. However, the primary lung adenocarcinoma did not response sensitively. This phenomenon may be explained by the molecular diversity of tumors in different locations [13].

The prognosis for lung cancer with metastasis to the esophagus is poor [14]. Therefore, the therapeutic strategy should be comprehensive and personalized. Our patient had an EGFR gene mutation in exon 19 and was treated with gefitinib. In June 2016, the patient’s brain metastases progressed. He took the original drug AZD9291 (osimertinib) on his own. It may be effective, as his headache symptoms subsided, although with a lack of clinical imaging evidence. Mounting evidence has now demonstrated that AZD9291 treatment is independently associated with longer overall survival in patients with a T790M mutation [15, 16]. In general, surgery is contraindicated in patients with organ metastases originating from lung cancer. However, some authors have reported prolonged survival in cases following management of oligometastasis of the bone, brain and small bowel [17] from lung cancer. There are no cases to date of patients who received local treatment for esophageal metastases from lung cancer. However, surgical intervention is often adopted when gastrointestinal metastases lead to continuous bleeding, obstruction or perforation [4].

## Conclusion

Esophageal metastases from lung cancer are extremely rare. However, clinicians should be aware of their occurrence. The diagnosis of esophageal metastasis is specific and is important to guide follow-up therapy. Comprehensive and personalized treatment may be beneficial in these patients. Molecular targeted therapy may be a reasonable choice in patients with EGFR mutations.

## Article highlights

Case characteristics

The patient was a 55-year-old man who presented to our hospital due to stimulated cough.

Clinical diagnosis

The patient was diagnosed with lung cancer.

Differential diagnosis

Mucosal biopsy from endoscopy was useful for differential diagnosis, and histological analysis revealed lung cancer.

Laboratory diagnosis

Laboratory findings revealed elevated tumor markers (CEA).

Imaging diagnosis

A computed tomography (CT) scan of the chest revealed a 48×42 mm defined tumor at the ascending left lung.

Pathological diagnosis

Pathological report of the tissue biopsy. Immunohistochemistry was positive for thyroid transcription factor 1 (TTF-1), cytokeratin 7 (CK-7) and napsin A. These results confirmed the diagnosis of esophageal metastasis from the lung.

Treatment

The patient underwent an EGFR gene testing of the primary lung cancer which harbored a 19th exon mutation. Gefitinib treatment and first-line chemotherapy based on docetaxel and cisplatin. After the chemotherapy, the patient then received the closed thoracic drainage and injected the lentinan and cisplatin (two cycles). Then the patient received six cycles of second-line chemotherapy with pemetrexed and cisplatin.

## Data Availability

We respect the patient’s rights to privacy, and to protect his identity, we do not wish to share our patient data. We presented in the manuscript all the necessary information about the case report. Raw data regarding our patient is in his admission file, a file that is strictly confidential, without the possibility of publishing raw data from it.

## Abbreviations

EUS: Endoscopic ultrasonography
TTF-1: Thyroid transcription factor 1
CK: Cytokeratin
CEA: Carcinoembryonic antigen
CT: Computed tomography
